# Phenolic acid phenethylesters and their corresponding ketones: Inhibition of 5‐lipoxygenase and stability in human blood and HepaRG cells

**DOI:** 10.1002/prp2.524

**Published:** 2019-09-13

**Authors:** Maroua Mbarik, Samuel J. Poirier, Jérémie Doiron, Ayyoub Selka, David A. Barnett, Marc Cormier, Mohamed Touaibia, Marc E. Surette

**Affiliations:** ^1^ Department of Chemistry and Biochemistry Université de Moncton Moncton NB Canada; ^2^ Atlantic Cancer Research Institute Moncton NB Canada

**Keywords:** 5‐lipoxygenase, caffeic acid phenethyl ester, inflammation, leukotriene

## Abstract

5‐lipoxygenase (5‐LO) catalyzes the biosynthesis of leukotrienes, potent lipid mediators involved in inflammatory diseases, and both 5‐LO and the leukotrienes are validated therapeutic targets. Caffeic acid phenethyl ester (CAPE) is an effective inhibitor of 5‐LO and leukotriene biosynthesis but is susceptible to hydrolysis by esterases. In this study a number of CAPE analogues were synthesized with modifications to the caffeoyl moiety and the replacement of the ester linkage with a ketone. Several new molecules showed better inhibition of leukotriene biosynthesis than CAPE in isolated human neutrophils and in whole blood with IC_50_ values in the nanomolar (290‐520 nmol/L) and low micromolar (1.0‐2.3 µmol/L) ranges, respectively. Sinapic acid and 2,5‐dihydroxy derivatives were more stable than CAPE in whole blood, and ketone analogues were degraded more slowly in HepaRG hepatocyte cultures than esters. All compounds underwent modification consistent with glucuronidation in HepaRG cultures as determined using LC‐MS/MS analysis, though the modified sinapoyl ketone (**10)** retained 50% of its inhibitory activity after up to one hour of incubation. This study has identified at least one CAPE analogue, compound **10**, that shows favorable properties that warrant further in vivo investigation as an antiinflammatory compound.

**Abbreviations**
5‐LO: 5‐lipoxygenase
CAPE: caffeic acid phenethyl ester
FLAP: 5‐lipoxygenase activating protein
LT: leukotriene
LTA_4_: leukotriene A_4_
LTB_4_: leukotriene B_4_
LTC_4_: leukotriene C_4_
PMNL: polymorphonuclear leukocyte
TPSA: topological polar surface area

## 1 INTRODUCTION

Leukotrienes (LTs) are a family of inflammatory mediators derived from arachidonic acid.^1^ 5‐Lipoxygenase (5‐LO) is the key enzyme in LT biosynthesis as it catalyzes the two‐step oxygenation of arachidonic acid forming LTA_4_.^2^, ^3^ LTA_4_ can then be hydrolyzed to LTB_4_ by a reaction catalyzed by LTA_4_ hydrolase or to the cysteinyl leukotrienes by LTC_4_ synthase.^4^, ^5^, ^6^ LTB_4_ is a potent leukocyte chemoattractant and cell activator. LTB_4_ and cysteinyl‐LT are implicated in vascular permeability and bronchoconstriction.^7^

**Figure 1:**
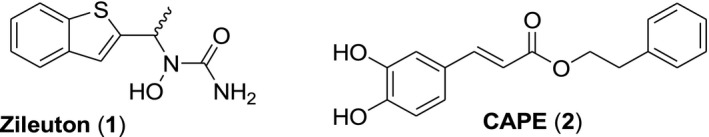
Structures of Zileuton (**1**) and CAPE (**2**)

Although inflammation is a defense mechanism vital to health, it also contributes to the development and progression of many chronic diseases.^8^, ^9^, ^10^, ^11^ 5‐LO and leukotrienes are intimately involved in the progression of inflammatory diseases like asthma, atherosclerosis and rheumatoid arthritis.^12^, ^13^, ^14^, ^15^ Several key proteins associated with LT biosynthesis and action, including 5‐LO, have been investigated and validated as antiinflammatory targets.^16^ To date, zileuton (**1**) (Zyflo^®^) (Figure 1) is the only drug which targets the leukotriene pathway through the inhibition of 5‐LO and is clinically approved in the United States for the chronic treatment of asthma.^17^ However, its use is limited due to hepatic side effects and an unfavorable pharmacokinetic profile.^18^, ^19^, ^20^ Hence the pursuit of new compounds targeting 5‐LO and the LT pathway remains an active area of research and clinical development.^21^

Several natural compounds have been investigated as potential 5‐LO inhibitors. Among these is caffeic acid phenethyl ester (CAPE) (**2**) (Figure 1), a bioactive component of honeybee propolis^22^ that has been reported to have beneficial health properties, including antiinflammatory activity.^23^, ^24^, ^25^, ^26^ Of importance, CAPE (**2**) inhibits LT biosynthesis in isolated human neutrophils with an IC_50_ (0.5 µmol/L) that is lower than that of zileuton (**1**) and that is similar (1.8 µmol/L) to that of zileuton (**1**) in whole blood.^27^, ^28^ This suggests that while remaining a potent inhibitor of LT biosynthesis, the susceptibility of CAPE (**2**) to modifications such as hydrolysis by esterases may reduce its potency in a physiological setting since caffeic acid itself is a poor 5‐LO inhibitor.^27^

Several analogues of CAPE (**2**) have been developed based on modifications of the caffeic moiety or of the ester linkage, and some have shown improved inhibition of leukotriene biosynthesis.^27^, ^29^, ^30^ In this study, CAPE (**2**) analogues in which the catechol moiety was modified and/or the ester bond was replaced with a ketone were investigated for their impact on the inhibition of leukotriene biosynthesis in human neutrophils and whole blood. Additionally, the stability of the compounds was evaluated in whole blood and in the human hepatocyte HepaRG cell model, and metabolites were identified using liquid chromatography‐mass spectrometry (LC‐MS/MS). In summary, sinapic acid and 2,5‐dihydroxy derivatives displayed a better inhibitory activity than caffeic acid derivatives and the ketone analogues displayed superior inhibition and stability. All compounds were modified by glucuronidation, however, the glucuronidated sinapic acid ketone analogue retained significant inhibitory activity.

## 2 METHODS

### 2.1 Synthesis of CAPE‐like analogues

Ester and ketone analogues of CAPE (**2**) were synthesized by one pot esterification or an aldol condensation, respectively, as previously reported.^29^, ^30^

### 2.2 Cell culture

HEK293 cells were obtained from ATCC (Manassas) and were cultured in Dulbecco’s Modified Eagle Medium (DMEM) supplemented with 10% fetal bovine serum. Cells were stably transfected with 5‐lipoxygenase and 5‐lipoxygenase activating protein (FLAP) as previously described.^31^

Undifferentiated cryopreserved HepaRG cells (BioPredic International) were seeded in 12 or 24 well plates at 1.1 × 10^5^ and 0.55 × 10^5^ cells per well, respectively. Cells were cultured for 14 days in Williams E‐medium supplemented with 1% penicillin‐streptomycin (10 000 U/mL), 10% fœtal bovine serum (FBS), 50 mg/L hydrocortisone hemisuccinate, 5 mg/L insulin and 2 mmol/L Glutamax, and the medium was renewed every 2‐3 days. After 14 days, the culture medium was replaced by the same formulation as above supplemented with 2% dimethyl sulfoxide (DMSO) to induce differentiation. Cells were considered differentiated and ready for experiments after 14 days of differentiation (28 days after seeding).

### 2.3 HEK293 cell stimulation and measurement of 5‐LO products

Transfected HEK293 cells were collected and resuspended in Hank's balanced salt solution (HBSS, Lonza) containing 1.6 mmol/L CaCl_2_ at a concentration of 5 × 10^5^ cells/mL, and were preincubated with each compound at concentration of 1 µmol/L for 5 minutes at 37°C. Cells were then stimulated for 15 minutes at 37°C with the addition of 10 µmol/L calcium ionophore A23187 (Sigma‐Aldrich) and 10 µmol/L arachidonic acid (Cayman Chemical).^28^ Stimulations were stopped by adding 0.5 volume of cold CH_3_OH:CH_3_CN (1:1) containing 100 ng/mL of prostaglandin B_2_ (PGB_2_) as internal standard. Samples were stored at −20°C and analyzed for 5‐LO products using reversed‐phase high‐performance liquid chromatography (RP‐HPLC) as described previously.^32^

### 2.4 Isolation and stimulation of polymorphonuclear leukocytes

Heparinized blood (see Ethics statement) was collected, centrifuged at 200*g* for 10 minutes to collect plasma, and erythrocytes were sedimented in dextran. PMNL were obtained after centrifugation on a lymphocyte separation medium cushion (density, 1.077 g/mL) (Wisent) at 900 g for 20 minutes, followed by hypotonic lysis to remove residual erythrocytes.^33^ PMNL were counted and resuspended at 10^7^ cells/mL in HBSS supplemented with 1.6 mmol/L CaCl_2_ and 0.3 U/mL adenosine deaminase (Sigma‐Aldrich). PMNL were preincubated with the test compounds or their diluent (0.1% DMSO) for 5 minutes at 37°C and were then stimulated with 1 µmol/L thapsigargin (Sigma‐Aldrich) for 15 minutes at 37°C.^27^ Reactions were stopped by adding two volumes of CH_3_OH:CH_3_CN (1:1) containing the internal standard PGB_2_ (100 ng/mL), and samples were processed for RP‐HPLC analysis of 5‐LO products.

In some experiments, platelets were removed from plasma by centrifugation at 2000*g* for 20 minutes, and test compounds were incubated in plasma for up to 60 minutes prior to addition to PMNL suspensions for subsequent stimulation and detection of 5‐LO products as described above.

### 2.5 Ex vivo whole blood stimulation

Blood was collected in tubes containing heparin as anticoagulant. Test compounds or their diluent control (DMSO, 0.1%) were added to 1 mL of heparinized blood at the indicated concentrations and incubated for 5 minutes in a water bath at 37°C. Stimulation was initiated with the addition of 125 μL of 40 mg/mL of opsonized zymosan, samples were then gently vortexed and incubated at 37°C for 30 minutes.^34^ Samples were then centrifuged at 960*g* for 10 minutes at 4°C, plasma was removed and added to tubes containing 4 volumes of CH_3_OH:CH_3_CN (1:1) containing 100 ng/mL of PGB_2_ as internal standard. Samples were stored overnight at −20°C, and then centrifuged at 3300*g* for 10 minutes. Samples were then purified on C18 cartridges, were eluted with CH_3_OH, dried under nitrogen, resuspended in 46% of CH_3_OH:CH_3_CN (1:1), and analyzed using RP‐HPLC as described above.

In some experiments, test compounds were incubated in blood for up to 120 minutes prior to stimulation with opsonized zymosan and detection of 5‐LO products as described above.

### 2.6 Incubation of test compounds with HepaRG cells

Differentiated HepaRG cells, plated in 24 well plates, were washed with hepatocyte suspension medium (Williams E‐medium without phenol red, supplemented with 2 mmol/L Glutamax and 12.5 mL HEPES) before addition of hepatocyte suspension medium (250 µL/well) containing (30 or 50 µmol/L) of the test compounds or their diluent. Cells were then incubated for 7.5, 15, 30, 60 or 90 minutes at 37°C after which the medium was removed.

In experiments where the test compounds incubated with HepaRG cells were to be measured, the cell layer was washed with 250 µL of hepatocyte suspension medium which was then pooled with the first medium. The mixture was then added to 250 µL of ice cold CH_3_OH:CH_3_CN (1:1, vol:vol) containing 10 µmol/L of phenethyl 3,4,5‐trimethoxycinnamate as an internal standard. The resulting solution was frozen at −80°C for 2 hour to precipitate any dissolved proteins, centrifuged at 1800*g* and the supernatant transferred to vials and stored at −20°C until HPLC analysis. Samples were then preconcentrated by in‐line solid phase extraction (Waters Oasis HLB 3.9 × 20 mm 15 µm Particle Size) followed by reverse phase HPLC (EMD Millipore Chromolith^®^ HighResolution RP‐18 endcapped 100 × 4.6 mm) on a linear gradient of 10% CH_3_CN in H_2_O + 0.1% formic acid to 100% CH_3_CN + 0.1% formic acid over 10 minutes at 2.2 mL/min with detection using a diode array at 270 nm (compounds **5**, **8**, Zileuton (**1**)) and 328 nm (CAPE (**2**), **7**, **9**, **10**) for detection and relative quantitation of test compounds.

In experiments where the inhibitory capacity of test compounds was to be determined after incubation with HepaRG cells, 125 µL of the hepatocyte suspension medium containing test compounds that had been collected at different time points from HepaRG cultures was added to 1 mL of heparinized blood. After a 5 minutes of preincubation, blood was then stimulated with opsonized zymosan and processed for HPLC analysis of 5‐LO metabolites as described above.

In other experiments, LC‐MS/MS analysis was performed to identify metabolites of test compounds that had been incubated with HepaRG cells. First, fifty micromolar standards of the test compounds were diluted 3000‐fold and analyzed using electrospray ionization mass spectrometry on a linear quadrupole ion trap (LTQ‐XL) from Thermo‐Fisher Scientific (San Jose). Full scan spectra were collected using both ionization polarities for confirmation of the intact molecular weight of each compounds based on the expected [M + H]^+^ and [M−H]^−^ pseudo‐molecular ions observed using mass spectrometry. The diluted standards were then subjected to reversed‐phase partition chromatography on an Altima C18 column produced by HiChrom and distributed by VWR (Mississsauga, ON). The LC system was a Dionex Ultimate 3000. The mass spectrometer electrospray source was used at 40 µL/min with a nitrogen sheath gas of 15, a spray voltage of ±4 kV, a capillary voltage of ±35 V, a tube lens of ±175 V and a capillary temperature of 160°C. Samples that had been collected from HepaRG cultures were purified on C18 cartridges and eluted with CH_3_OH. For the first injection of extracted samples, the MS method consisted of a repeated loop of full scan (*m*/*z* 100‐800), product ion scan based on the intact mass determined from infusion of the standards and one product ion scan. Subsequent injection of all samples from both time points were performed with individualized scanning functions for each treatment in negative ion mode only. The scan functions consisted of a cycle of full scan (ie, *m*/*z* −100 to −800), a product ion scan of the input compound as well as a product ion scan of the identified metabolite.

### 2.7 Docking

Docking of all inhibitors into the active site of the crystal structures of human 5‐LO (PDB code: 3O8Y, 2.39 A resolution)^35^ as well as the calculations of the affinities of the test molecules was performed with the AutoDock vina.^36^

### 2.8 Statistics

Statistical analysis and graph design were performed with GraphPad Prism 6 software (GraphPad Software). IC_50_ values were calculated from a sigmoidal concentration‐response curve‐fitting model. Half‐life time values were calculated from exponential decay curves. IC_50_ and half lifetime values are expressed as means with 95% confidence intervals. One‐way ANOVA or two‐way ANOVA with Dunnett's multiple comparison test was performed to determine significant difference from controls (*P* < .05). Data are expressed as means ± SEM.

## 3 RESULTS

### 3.1 Biosynthesis of 5‐LO products

**Figure 2:**
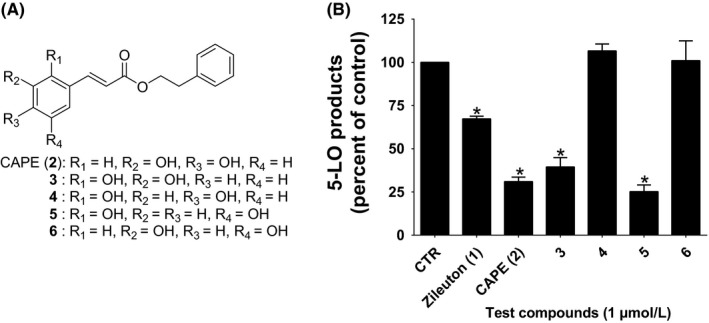
The structures of the tested compounds (**A**) and their effects on the biosynthesis of 5‐LO products in transfected HEK293 cells (**B**). HEK293 cells expressing 5‐LO and FLAP were incubated in presence of the indicated compounds (1 μmol/L) or their diluent (Control, 0.1% DMSO) for 5 minutes, followed by the addition of 10 μmol/L calcium ionophore A23187 and 10 μmol/L arachidonic acid for 15 minutes. Reactions were then stopped, and samples were processed for analysis of 5‐LO products using RP‐HPLC. Total 5‐LO products measured represent the sum of LTB_4_, its trans isomers and 5‐hydroxyeicosatetraenoic acid. Data are expressed as means ± SEM of at least three independent experiments. *Difference from control as determined using one‐way ANOVA with Dunnett's multiple comparison test (*P* < .05)

Elimination of the catechol or modifications of the functional groups on the caffeic moiety of CAPE has resulted in compounds that poorly inhibit LT biosynthesis,^27^, ^29^ with the exception of the sinapic acid (2,4‐dimethoxy, 3‐hydroxy) derivative that results in better inhibitory activity than CAPE (**2**).^29^ Therefore, a first series of experiments used a HEK293 cell model that ectopically expresses 5‐LO and FLAP to screen other dihydroxy isomers of CAPE to determine if the positions of the hydroxyl groups are of importance. The HEK293 cells were incubated with 1 μmol/L of CAPE (**2**) or its dihydroxy isomers **3**, **4**, **5** and **6** (Figure 2A) prior to stimulation. Figure 2B shows that the positive control, zileuton (**1**), was less effective than CAPE (**2**), but that compounds **3** and **5** exhibited very good inhibitory activity similar to that of CAPE (**2**), whereas compounds **4** and **6** were without measurable effect. Therefore, the position of the two hydroxyl groups is of importance for 5‐LO inhibition.

**Figure 3:**
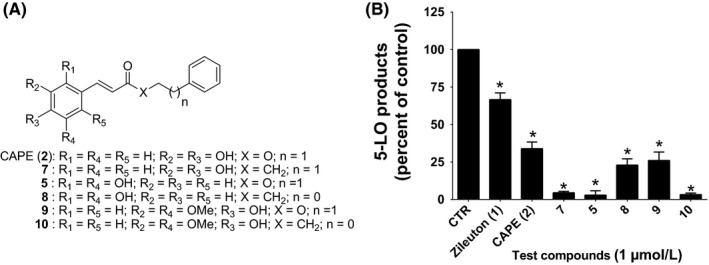
The structure of the test compounds and their effect on the biosynthesis of 5‐LO products in PMNL. (A) The structures of CAPE (**2**) and its analogues investigated in this study. (B) Isolated human PMNL were incubated in presence of test compounds (1 μmol/L) or their diluent (Control, 0.1% DMSO) for 5 minutes. PMNL were stimulated with 1 μmol/L of thapsigargin for 15 minutes at 37°C. The stimulation was then stopped, and samples were processed for analysis of 5‐LO products using RP‐HPLC. Total 5‐LO products measured represent LTB_4_, its trans isomers, 20‐OH‐LTB_4_, 20‐COOH‐LTB_4_ and 5‐hydroxyeicosatetraenoic acid. Data are expressed as means ± SEM of at least three independent experiments. *Difference from control, as determined using one‐way ANOVA with Dunnett's multiple comparison test (*P* < .05)

After screening in the HEK293 cell model, in a second set of experiments the inhibitory activity on LT biosynthesis of the three compounds showing the best inhibitory activity, CAPE (**2**) and **5**, as well as sinapic acid phenethylester (**9**),^29^ was compared in stimulated human PMNL. Since ketone derivatives of CAPE (**2**) and compound **5** (compounds **7** and **8**, respectively) were previously shown to also exhibit significant inhibition,^30^, ^37^ a ketone derivative of **9** (compound **10**) (Figure 3A) was also synthesized and tested for inhibitory activity. All six test compounds showed a significant inhibition of LT biosynthesis in human PMNL, with compounds **5**, **7** and **10** showing a near complete inhibition at the tested concentration (Figure 3B).

Since all six selected test compounds showed significant inhibition at a concentration of 1 μmol/L, the compounds were screened further in concentration‐response studies to better characterize their relative inhibitory capacities in stimulated PMNL and in whole blood (Table 1). All compounds were assayed at concentrations of 0, 0.1, 0.3, 1 and 3 μmol/L in PMNL and 0, 0.1, 0.3, 1, 3 and 10 μmol/L in whole blood in order to obtain dose‐response curves, from which IC_50_ values were calculated.

**Table 1:** Calculated IC_50_ values of selected compounds for the inhibition of 5‐LO product biosynthesis in human PMNL and in whole blood

Compounds	PMNL IC_50_ (µmol/L)	Whole blood IC_50_ (µmol/L)
Zileuton (**1**)	2.30 (1.95‐2.72)	1.93 (1.57‐2.37)
CAPE (**2**)	0.97 (0.85‐1.10)	3.58 (3.03‐4.24)
**7**	0.52 (0.45‐0.61)	2.30 (1.72‐3.08)
**5**	0.32 (0.29‐0.36)	1.00 (0.88‐1.14)
**8**	0.51 (0.44‐0.58)	1.15 (0.99‐1.34)
**9**	0.29 (0.27‐0.32)	1.91 (1.51‐2.41)
**10**	0.41 (0.36‐0.46)	1.20 (0.98‐1.46)

Note

Values are means (95% confidence interval) from at least three independent experiments.

In PMNL, all compounds outperformed CAPE (**2**) and possessed IC_50_ values that were significantly lower than the clinically approved 5‐LO inhibitor Zileuton (**1**). However, in whole blood, although Zileuton (**1**) had a lower IC_50_ value than CAPE (**2**), compounds **7** and **9** were similar to zileuton, while compounds **5**, **8** and **10** all showed lower IC_50_ values than zileuton.

### 3.2 Molecular docking

Docking of all inhibitors into the active site of the crystal structures of human 5‐LO (PDB code: 3O8Y, 2.39 A resolution)^35^ was performed with AutoDock Vina. As shown in Table 2, all compounds showed affinity for the active site, with compound **8** scoring the best affinity with a docking energy of −9.1 kcal/mol. A hydrogen bond with His 600 (OH ··· N: 3.04 Å) was detected by LigPlot+. Compound **8** also had π‐π interactions with His 367 and His 372, both of which coordinate the iron atom^35^ while His372 may act as a replaceable coordinating ligand for the iron atom.^38^ Compound **5** (−8.8 kcal/mol) had interactions that were very similar to compound **8** forming a hydrogen bond with His 600 (OH ··· N: 3.22 Å) and a π‐π interaction with His 372.

**Table 2:** Molecular modeling results showing the affinity of the test compounds for the active site of 5‐LO, π‐π interactions and hydrogen bonds

Compounds	Affinity (kcal/mol)	π‐π Interactions	H‐Bonds	Distance (Å)
(*R*)‐Zileuton (**1**)	−6.6	Phe421	Leu420 x 2, Asn425	2.50, 3.15, 3.29
(*S*)‐Zileuton (**1**)	−6.5	–	Leu420 x 2, Ala424, Phe421	2.81, 3.09, 3.34, 2.97
CAPE (**2**)	−8.8	His372	Leu420	
**7**	−8.7	–`	Phe177, His367	
**5**	−8.8	His372	His600	3.22
**8**	−9.1	His367, His372	His600	3.04
**9**	−8.7	His372	–	
**10**	−8.2	His372	–	

The remaining ligands had a similar affinity energy, with the exception of Zileuton (**1**) that showed lower affinity for both active enantiomers. CAPE (**2**) (−8.8 kcal/mol) formed a hydrogen bond with Leu 420 (OH ··· O: 3.07 Å) and a π‐π interaction with His372. Compound **7** (−8.9 kcal/mol) underwent pi‐pi interactions with His367 and was the only tested ligand that showed a π‐π interaction with Phe177 which may play a part in product specificity.^39^ Compounds **9** (−8.7 kcal/mol) and **10** (−8.7 kcal/mol) had pi‐pi interactions with His 372. Overall, all molecules showed affinity for the active site although the calculated affinities do not appear to correlate with IC_50_ data.

All tested ligands had the same orientation (data not shown) with the substituted benzene portion of the molecules pointing towards the end of the cavity and the nonsubstituted portion approaching the iron atom. The ligands had a slightly different pose from each other. Compounds **9** and **10** were partially superimposed on the substituted ring but they differed after the double bond in the connecting chain due the greater flexibility allowed by the missing ester group which is substituted by a carbonyl group in compound **10**.

### 3.3 In silico* physicochemical properties and drug‐likeness evaluation*

The physicochemical properties of the selected compounds were evaluated to determine if they are within the Lipinski's Rule of Five, which is important for pharmacokinetics and drug development.

For this purpose, physicochemical and ADME (absorption, distribution, metabolism, and excretion) properties were calculated using the SwissADME (a free web tool to evaluate pharmacokinetics, drug‐likeness and medicinal chemistry friendliness of small molecules) (Table 3). Compounds obeying at least three of the four criteria are considered to adhere to Lipinski Rule,^40^ and all tested compounds were compatible with Lipinski Rule. Other properties of interest are the number of rotatable bonds and the topological polar surface area (TPSA). Compounds with many rotatable bonds (>10) have been associated with poor oral bioavailability^41^ while compounds with a low TPSA (<140 Å2) tend to have higher oral bioavailability.^41^, ^42^ All tested compounds showed favorable PSA and number of rotatable bonds.

**Table 3:** Absorption, distribution, metabolism, and excretion (ADME) profile of molecules of interest

	Physicochemical properties				Lipophilicity		Pharmaco‐kinetics	
	MW (g/mol)	ROTB (n)	HBA (n)	HBD (n)	TPSA (Å)	CLog P_o/w_	GIA	BBBP
Rule	<500	≤10	<10	<5	≤140	<5	–	–
Zileuton (**1**)	236	3	2	2	94.80	1.81	High	Yes
CAPE (**2**)	284	6	4	2	66.76	3	High	Yes
**7**	282	6	3	2	57.53	3.37	High	Yes
**5**	284	6	4	2	66.76	3.01	High	Yes
**8**	268	5	3	2	57.53	3	High	Yes
**9**	328	8	5	1	64.99	3.35	High	Yes
**10**	312	7	4	1	55.76	3.42	High	Yes

Abbreviations: BBBP, blood brain barrier permeation; CLog Po/w, logarithm of compound partition coefficient between n‐octanol and water; GIA, gastrointestinal absorption; HBA, hydrogen bond acceptors; HBD, hydrogen bond donors; MW, molecular weight; n, number; ROTB, rotatable bonds; TPSA, topological polar surface area.

### 3.4 Stability of test compounds in plasma and whole blood

**Figure 4:**
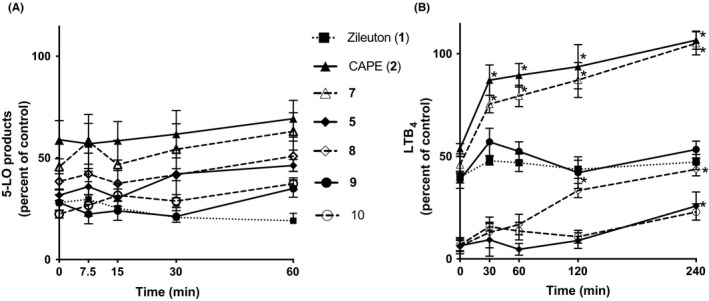
Stability of the test compounds for the inhibition of the biosynthesis of 5‐LO products following incubation in plasma or in whole blood. (A) Test compounds were incubated (30 µmol/L) in human plasma for the indicated times at 37°C. Plasma (50 µL) containing the test compounds was then added to 450 µL of PMNL (10^7^ cells/mL) which were then stimulated with 1 μmol/L of thapsigargin for 15 minutes at 37°C. The stimulation was then stopped, samples were processed, and 5‐LO products were measured using RP‐HPLC. (B) Whole blood was incubated in the presence of 3 μmol/L of test compounds or with their diluent (control, DMSO 0.1%) for the indicated times and was then stimulated with opsonized zymosan (4 mg/mL) for 30 minutes at 37°C. Blood was then centrifuged and plasma was collected and added to 1.2 mL of CH_3_OH:CH_3_CN (1:1). Samples were processed for analysis using RP‐HPLC for LTB_4_ quantification. All data are expressed as means ± SEM of at least three independent experiments. *Difference from time = 0, as determined using One‐way ANOVA with Dunnett's multiple comparison test (*P* < .05)

The stability of the inhibitory activity of the test compounds was evaluated in human plasma and in whole blood. Firstly, compounds were incubated at 37°C for various times in human plasma and were then evaluated for their ability to inhibit the stimulated biosynthesis of 5‐LO products in human PMNL. Figure 4A shows that all compounds had a steady inhibitory effect over time which suggests that the compounds are stable in plasma. A second series of experiments was performed in whole blood by incubating the test compounds or their diluent (DMSO 0.1%) for various times in whole blood prior to stimulation with zymosan. Unlike human plasma results, the inhibitory effect of CAPE (**2**) and compound **7**, the two compounds with catechol groups, decreased over time with a complete loss of inhibitory activity after 240 minutes of incubation (Figure 4B). The inhibitory activity of all other compounds remained quite stable over time, with only compound **8** showing approximately 25%‐35% loss of activity after 2‐4 hours of incubation in blood.

### 3.5 Stability of test compounds in HepaRG cell culture

HepaRG cells can be differentiated into hepatocytes and biliary‐like epithelial cells and maintain liver functions such as expression of drug metabolizing enzymes and transporters.^43^, ^44^ Therefore, this is a valuable in vitro model to assess the potential hepatic stability of experimental compounds. To study the kinetics of test compound stability and its effects on the biosynthesis of LTB_4_, the test compounds were incubated with HepaRG cells and the culture medium was then collected at different times to measure their remaining presence and the residual capacity to inhibit the biosynthesis of 5‐LO products.

**Figure 5:**
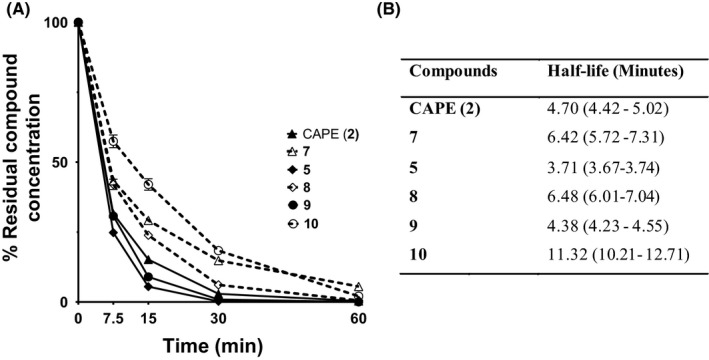
Kinetics of test compound disappearance in HepaRG cell culture. (A) HepaRG cells were incubated in 250 µL of hepatocyte suspension medium containing 30 µmol/L of test compounds for the indicated times at 37°C. Supernatants were removed and added to 250 µL of ice cold CH_3_OH:CH_3_CN (1:1 vol:vol) containing 10 µmol/L of phenethyl 3,4,5‐trimethoxycinnamate as an internal standard. The cell layer was washed with 250 µL of hepatocyte suspension medium and added to the above mixture. Samples were centrifuged at 1900*g* and the supernatants were collected for HPLC analysis as described in the Methods. Values are means ± SEM. (**B**) Calculated half‐lives of the test compounds in HepaRG cell cultures. Values are means (95% confidence interval). All data are from four independent experiments

Based on the relative peak area for each compound compared to unmetabolized starting material (t = 0) following separation by HPLC, peaks associated with all three ester‐linked compounds **2**, **5** and **9** disappeared from HLPC chromatograms more quickly (Figure 5A) and exhibited shorter half‐lives (Figure 5B) than their ketone counterparts. The sinapic acid ketone analogue **10** had a significantly greater half‐life than all other analogues.

**Figure 6:**
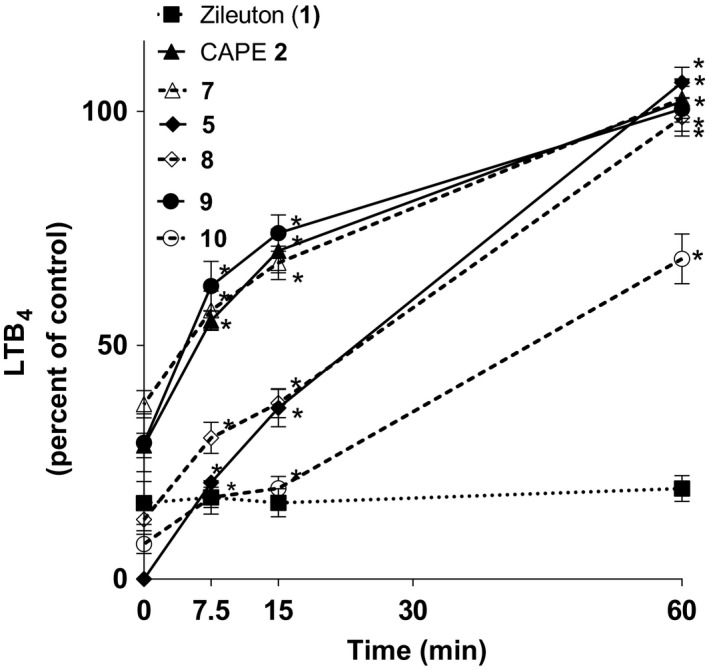
Stability of the test compounds for the inhibition of the biosynthesis of 5‐LO products following incubation with HepaRG cells. HepaRG cells were incubated with 500 µL of hepatocyte suspension medium containing 50 µmol/L of test compounds for the indicated times at 37°C. Hepatocyte suspension medium (125 µL) was then removed and added to 1 mL of blood that was then incubated for 5 minutes and then stimulated with opsonized zymosan (4 mg/mL) for 30 minutes at 37°C. Blood was then centrifuged and plasma was collected and added to 1.2 mL of CH_3_OH:CH_3_CN (1:1). Samples were processed for analysis using RP‐HPLC for LTB_4_ quantification. Data are expressed as means ± SEM of at least three independent experiments. *Difference from time = 0, as determined using One‐way ANOVA with Dunnett's multiple comparison test (*P* < .05)

The loss of inhibitory activity of the test compounds (Figure 6) followed a similar pattern to that of their disappearance from the cell culture medium. After 15 minutes most compounds lost some inhibitory activity against 5‐LO product biosynthesis, although the loss of biological activity was not as apparent as the loss of peak height seen in Figure 5. After 60 minutes CAPE (**2**), compounds **7**, **5**, **8** and **9** had lost all their inhibitory activity (Figure 6). However, compound **10** lost its inhibitory activity much more slowly over time and maintained significant inhibition of the biosynthesis of 5‐LO products after 60 minutes despite its complete disappearance from culture medium as shown in Figure 5. The comparator compound zileuton inhibited the 5‐LO product biosynthesis in a steady manner over time.

### 3.6 Identification of metabolites following incubation of test compounds in HepaRG cell culture

The goal of the next set of experiments was to identify using LC‐MS/MS the metabolites formed following incubation with HepaRG cells. Firstly, the retention time and fragmentation pattern were determined using negative ion and positive ion LC‐MS/MS for each test compound prior to incubation with cells. Table S1 shows that the expected mass for each compound was obtained using LC‐MS and also shows the mass of the fragments obtained and the HPLC retention times. HepaRG cells were then incubated in the presence of each test compound for 7.5 and 60 minutes at 37°C and the supernatants were analyzed using LC‐MS/MS.

**Figure 7:**
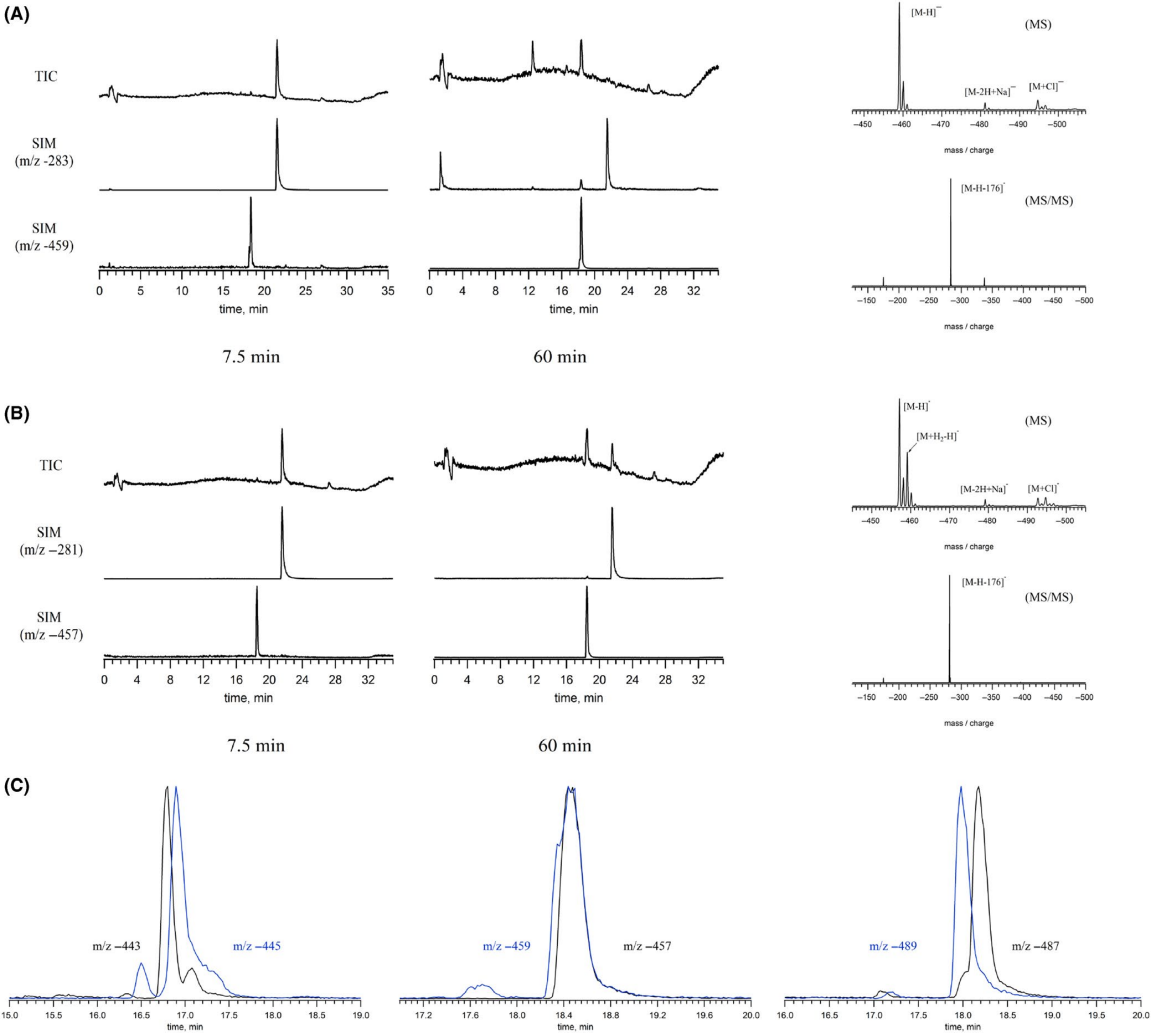
Metabolite identification using negative ion LC‐MS/MS in HepaRG cell culture. HepaRG cells were incubated in hepatocyte suspension medium containing 50 µmol/L of CAPE (**2**) (A) or Compound **7** (B) for 7.5 and 60 minutes at 37°C. The supernatant was removed, added to one volume of methanol and samples were subjected to off‐line C18 solid phase extraction prior to injection on the LC‐MS/MS platform for analysis in negative ion mode. Left panels show total ion count (TIC) chromatograms as well as single ion monitoring (SIM). Right panels show the MS and MS/MS of the generated metabolites detected at 60 minutes. (**C**) SIM chromatograms of [M‐H+]^‐^ and [M + H_2_‐H+]^‐^ ions for the metabolites derived from the three ketone compounds **10**, **8** and **7**

Figure 7A shows the negative ion LC‐MS analysis of CAPE (**2**) following incubation with HepaRG cells for 7.5 and 60 minutes. At 7.5 minutes, the total ion chromatogram (TIC) shows one peak that corresponds to the retention time and mass of CAPE (m/z‐283), however after 60 minutes a second peak appears with m/z‐459. Single ion monitoring (SIM) of the second peak shows that the compound with *m*/*z*‐459 was also present at 7.5 minutes. The fragmentation of this second peak by MS/MS shows a fragment with a mass of [M‐H‐176]^−^ that corresponds to the expected mass of glucuronic acid. A similar profile was also seen for the ketone analogue of CAPE (**2**), compound **7** (Figure 7B). The results of the mass spectral analyses of all test compounds are summarized in Table S1B.

Additionally, the presence of [M + H_2_‐H]^+^ ions seen in Figure 7B suggests the hydrogenation of a double bond. In fact, this pattern was observed for all ketone compounds **7**, **8** and **10** that had been incubated with HepaRG cells but not for their respective esters. The SIM chromatograms obtained with the respective metabolite's mass and that of the M + H_2_ ions show that the peaks are resolved for compounds **8** and **10**, but not **7** (Figure 7C). This suggests that these compounds undergo α,β‐unsaturated ketone reduction in these culture conditions. Based on the relative peak areas, a significant proportion of the molecules undergo hydrogenation.

## 4 DISCUSSION

5‐Lipoxygenase and its products, the leukotrienes, are intimately involved in the inflammatory response and have been targeted for the treatment of asthma for over two decades with the development of the 5‐LO inhibitor zileuton (Zyflo^®^) and the cys‐leukotriene‐1 (Cys‐LT1) receptor antagonists montelukast (Singulair^®^) and zafirlukast (Accolate^®^). More recently, 5‐LO has been identified as a potential therapeutic target for conditions such as rheumatoid arthritis,^45^, ^46^ atherosclerosis,^47^, ^48^, ^49^ Alzheimer's disease^50^ and leukemia stem cells.^51^, ^52^ However, zileuton has poor pharmacokinetic properties and liver toxicity issues, whereas the Cys‐LT1 receptor antagonists only target the cys‐LTs and not the LTB_4_ receptors that play an important role in several pathologies. Therefore, there is interest for the development of effective 5‐LO inhibitors.^21^

In this study, analogues of the natural polyphenolic compound CAPE (**2**) were synthesized and evaluated as 5‐LO inhibitors. CAPE is a potent 5‐LO inhibitor with IC_50_ values in the high nmol/L to low μmol/L range in isolated human neutrophils and in whole human blood, respectively.^27^ However, its stability as a drug candidate has been questioned partly because of its susceptibility to esterases^53^ that catalyze its hydrolysis to the much less active caffeic acid.^27^ The analogues described in this study include ketones that are not susceptible to esterase action, as well as analogues of the caffeoyl moiety of the molecule that show enhanced inhibitory activity toward the biosynthesis of 5‐LO products. Some of these analogues were shown to be more resistant to degradation and to loss of inhibitory activity following incubation in HepaRG cell culture and in whole human blood.

With regard to the caffeoyl moiety of CAPE (**2**), it was previously shown that cinnamic acid phenethyl ester, devoid of the vicinal hydroxyl groups, and various monohydroxy derivatives of CAPE (**2**) do not inhibit LT biosynthesis at concentrations up to 10 µmol/L.^27^, ^29^ Similarly, mono, di‐ and tri‐methoxy derivatives of CAPE (**2**) are also without inhibitory activity.^29^ The only other substitution previously tested that results in inhibitory activity similar to or better than CAPE (**2**) was the sinapic acid (3,5‐dimethoxy, 4‐hydroxy) derivative, compound **9**.^29^ This study provides additional information regarding the impact of substitutions on the caffeoyl moiety. Unlike CAPE (**2**), the 2,4‐dihydroxy and the 3,5‐dihydroxy compounds **4** and **6**, respectively, were without inhibitory activity at the tested concentrations, whereas the 2,5‐dihydroxy compound **5** showed superior inhibition of 5‐LO product biosynthesis compared to CAPE (**2**) in isolated PMNL and in whole blood. In fact, compound **5** was equivalent to the sinapic acid derivative **9** in isolated PMNL and showed superior inhibition in whole blood. Therefore, the position of the hydroxyl groups on the phenol is critical to the capacity to inhibit 5‐LO product biosynthesis.

Although CAPE (**2**) and compounds **5** and **9** exhibited important inhibitory activity in short‐term incubations in isolated PMNL and whole blood, previous studies showed that CAPE (**2**) is rapidly hydrolyzed to caffeic acid in rat plasma (but not in human plasma) by a carboxylesterase, and oral administration to rats also results in excretion of caffeic acid as the major metabolite in the urine.^53^, ^54^, ^55^ Since an effective drug candidate should not be enzymatically labile in plasma, CAPE analogues were synthesized with structural modifications of the ester bond. It was previously shown that an amide analogue of CAPE inhibited LT biosynthesis and 5‐LO activity, though with reduced potency compared to CAPE.^27^ However, ketone derivatives of CAPE (**2**) have shown inhibition of 5‐LO product biosynthesis in human PMNL that appear to be superior to that of CAPE (**2**).^37^ Therefore, ketone derivatives of the most effective esters were synthesized with the speculation that they should be more potent and more stable than their ester analogues. As expected, the ketone derivatives of CAPE (**2**) were generally as good or better inhibitors of 5‐LO product biosynthesis as their ester analogues in both isolated PMNL and whole blood in short‐term incubations. However, although the inhibitory capacity of all compounds appeared to be stable in plasma, only CAPE (**2**) and compound **7** rapidly lost their ability to inhibit leukotriene biosynthesis following a preincubation in blood suggesting that the vicinal hydroxyl groups are susceptible to modification in whole blood.

Human HepaRG cells have been used in many studies as a valuable alternative to ex vivo cultivated primary human hepatocytes to evaluate drugs and perform drug metabolism studies. In such experiments HepaRG cells are differentiated into a hepatocyte‐like morphology while conserving the expression of cytochrome P450 enzymes, transporter proteins, and transcription factors.^43^, ^44^ The incubation of all test compounds with HepaRG cells resulted in the production of glucuronidated analogues, consistent with the known susceptibility of hydroxyl groups of phenolic compounds to sulfation and glucuronidation,^56^, ^57^, ^58^ although no sulfated analogues were detected. Interestingly, the half‐life of the parent compounds in HepaRG cultures varied as the ketone derivatives all showed longer half‐lives than the ester compounds, with the sinapic acid ketone compound **10** showing a significantly greater half‐life than all other compounds. Although these in vitro results cannot predict with certainty which compounds will exhibit acceptable pharmacokinetic profiles, these results indicate that compound **10** is the preferred candidate for further in vivo evaluation. Moreover, compound **10** was the only molecule that retained inhibitory activity after 60 minutes in HepaRG culture despite the complete disappearance of the parent compound on HPLC chromatograms, suggesting that the glucuronide conjugate product exhibits inhibitory activity. These observations coupled with its near complete retention of inhibitory activity in whole blood after a 4‐hour incubation and its excellent IC_50_ value further support the continued investigation of this compound for subsequent in vivo evaluation.

Another observation following LC‐MS analysis of HepaRG cultures was the presence of [M‐H]^+^ and [M + H_2_‐H]^+^ ions in incubations that contained the ketone compounds but not the esters. The ions were resolved chromatographically and are likely the results α,β‐unsaturated ketoalkene reductase that has been documented in the liver.^59^, ^60^, ^61^ Importantly, the saturated form of CAPE (**2**)^29^ was previously shown to be as effective for the inhibition of 5‐LO product biosynthesis, thus the reduction of the α,β‐unsaturated ketoalkene in the liver is unlikely to impact on the capacity of the ketone compounds to inhibit 5‐LO product biosynthesis.

All tested compounds showed favorable physicochemical and ADME properties that are compatible with favorable pharmacokinetic and drug‐likeness properties, as well as characteristics that are consistent with oral bioavailability. Indeed, all compounds adhered to Lipinski's rule of 5 indicating that they do not trigger a computational alert for absorption or permeation that would suggest poor bioavailability.^40^ Compounds that trigger the alert are likely to be troublesome in subsequent in vivo studies. In a preliminary evaluation of the molecular interactions between selected compounds and 5‐lipoxygenase, all compounds showed a stronger affinity to 5‐LO than zileuton. Furthermore, several interactions were identified that are consistent with the inhibition of enzyme activity such as interactions with His 600 that may be necessary for the positioning of the arachidonic acid substrate (compounds **5** and **8**), π‐π interactions with Phe177 (compound **7**) which may play role in product specificity^39^ and π‐π interactions with His 372 (CAPE (**2**), **5**, **9**, **8** and **10**) that suggest a good position to coordinate the iron atom or, at a minimum, block some access to the binding site cavity.

Although CAPE has been shown to be an excellent inhibitor of 5‐LO product biosynthesis, a series of structure‐activity relationship studies were undertaken to develop new compounds that possess enhanced biological activity.^27^, ^28^, ^29^, ^30^, ^37^ Predicting the best performing analogues has not been apparent. For example, the relation between the presence and position of dihydroxy groups or methoxy groups and inhibitory activity is critical but was not predictable. Similarly, different structural analogues of the ester moiety show significantly different inhibitory activity, and possessing radical scavenging activity comparable to that of CAPE does not translate into good 5‐LO inhibition. Amongst the numerous CAPE analogues that have been evaluated, the 2,5‐dihydroxy compounds (**5 and 8)** and the sinapic acid derivatives (**9** and **10**) described in this study are those that have shown the best inhibitory activity in isolated PMN and in whole blood. This study was the first to evaluate the biological stability of these analogues. While ketone derivatives showed better stability than esters, sinapic acid phenethyl ketone (**10**) showed the longest half‐life and was the only molecule that maintained inhibitory activity in hepatocyte cultures. Overall, the excellent inhibition of 5‐LO product biosynthesis in a complex matrix combined with its better stability and favorable physicochemical and ADME properties suggest that compound **10** is a good candidate for continued development in preclinical models of inflammatory diseases.

## DISCLOSURE

Authors ME Surette and M Touaibia are the inventors of a patent application that describes some of the compounds reported in the current study.

## AUTHOR CONTRIBUTIONS

Participated in research design: Surette, Mbarik and Poirier;

Performed experiments: Mbarik, Poirier, Doiron, Barnett, and Cormier;

Contributed new reagent or analytic tools: Selka, Touaibia;

Performed data analysis: Surette, Mbarik, Doiron, Barnett, and Touaibia;

Wrote or contributed to the writing of the manuscript: Mbarik, Touaibia, Barnett, and Surette.

## FUNDING INFORMATION

This work was supported by grants from the National Sciences and Engineering Research Council of Canada; the New Brunswick Health Research Foundation; the New Brunswick Innovation Foundation.

## ETHICS

This study was approved by the Université de Moncton institutional Review Committee for Research involving human subjects (approval number 1314‐029). All subjects provided written informed consent prior to their participation in the study.

## Data Availability

The data that support the findings of this study are available from the corresponding author upon reasonable request.
