# Supplementary material for: Phenolic acid phenethylesters and their corresponding ketones: Inhibition of 5‐lipoxygenase and stability in human blood and HepaRG cells
Source: Pharmacol Res Perspect. 2019 Sep 13;7(5):e00524. doi: 10.1002/prp2.524 (PMC6743424; doi:10.1002/prp2.524)
Supplement: Supplementary file 1 [file PRP2-7-e00524-s001.docx]

**Supplemental Table S1.** Summary results of mass spectrometry analysis of test compounds and their metabolites following incubation with HepaRG cells. (FWHM = full width at half maximum height)

| **A** | **Compounds** | | | | | | |
| --- | --- | --- | --- | --- | --- | --- | --- |
|  | | CAPE (**2**) | **7** | **5** | **8** | **9** | **10** |
| \| **Mass (-ve)** \| \| --- \| | | 283 | 281 | 283 | 267 | 327 | 311 |
| **Fragment (-ve)** | | 179 | 177 | 161 | 135 | 312 | 296 |
| **Mass (+ve)** | | 285 | 283 | 285 | 269 | 329 | 313 |
| **Fragment (+ve)** | | 105 | 147 | 105 | 165 | 207 | 295 |
| **Retention Time (min)** | | 21.5 | 22.4 | 20.7 | 20.0 | 23.0 | 22.4 |
| **Retention Factor, k'** | | 11.6 | 12.2 | 11.2 | 10.8 | 12.5 | 12.2 |
| **FWHM, (s)** | | 10.2 | 10.2 | 8.4 | 11.4 | 7.2 | 7.2 |
| **B** | **Metabolites** | | | | | | |
|  | | CAPE (**2**) | **7** | **5** | **8** | **9** | **10** |
| \| **Mass (-ve)** \| \| --- \| | | 459 | 457 | 459 | 443 | 503 | 487 |
| **Fragment (-ve)** | | 283 | 281 | 283/175 | 267/175 | 327 | 311 |
| **Mass (+ve)** | | 461 | 459 | 461 | 445 | 505 | 489 |
| **Fragment (+ve)** | | 285 | 283 | 285 | 269 | 329 | 313 |
| **Retention Time (min)** | | 18.3 | 18.4 | 17.4 | 16.8 | 18.7 | 18.4 |
| **Retention Factor, k'** | | 9.8 | 9.8 | 9.2 | 8.9 | 10.0 | 9.8 |
| **FWHM, (s)** | | 10.2 | 10.2 | 9.0 | 7.8 | 9.0 | 9.0 |
